# The frail-LESS (LEss sitting and sarcopenia in frail older adults) remote intervention to improve sarcopenia and maintain independent living via reductions in sedentary behaviour: findings from a randomised controlled feasibility trial

**DOI:** 10.1186/s12877-024-05310-9

**Published:** 2024-09-09

**Authors:** Daniel P. Bailey, Jamie H. Harper, Cherry Kilbride, Laura J. McGowan, Christina Victor, Marsha L. Brierley, Angel M. Chater

**Affiliations:** 1 Centre for Physical Activity in Health and Disease, Brunel University London, Kingston Lane, UB8 3PH Uxbridge, UK; 2 Division of Sport, Health and Exercise Sciences, Department of Life Sciences, Brunel University London, UB8 3PH Uxbridge, UK; 3 Division of Physiotherapy and Physician Associates, Department of Health Sciences, Brunel University London, UB8 3PH Uxbridge, UK; 4 NIHR Policy Research Unit in Behavioural Science – Population Health Sciences Institute, Faculty of Medical Sciences, Newcastle University, Newcastle upon Tyne, UK; 5 Division of Global Public Health, Brunel University London, UB8 3PH Uxbridge, UK; 6 Institute for Sport and Physical Activity Research, University of Bedfordshire, Polhill Avenue, MK41 9EA Bedford, UK; 7 Centre for Behaviour Change, University College London, WC1E 7HB London, UK

**Keywords:** Sedentary behaviour, Sitting, Frailty, Sarcopenia, Physical function, Feasibility study, Behaviour change

## Abstract

**Background:**

Sarcopenia leads to functional disability, dependence in activities of daily living (ADL), and is a key contributor to frailty. Reducing and breaking up sedentary time is associated with improved sarcopenia and frailty-related outcomes. The aim of this study was to determine the feasibility of delivering and evaluating a remote sedentary behaviour intervention to improve sarcopenia and independent living in older adults with frailty.

**Methods:**

A two-arm randomised controlled feasibility trial was conducted with a target of 60 older adults (mean age 74 ± 6 years) with very mild or mild frailty. Participants were randomised to the Frail-LESS (LEss Sitting and Sarcopenia in Frail older adults) intervention or usual care control group for six months. The intervention included tailored feedback on sitting, standing and stepping; an education workbook that included goal setting and action planning; one-to-one health coaching; peer support; and a wearable device to self-monitor sedentary behaviour. Participant recruitment (percentage of eligible individuals recruited), retention and data completion rates were used to assess trial feasibility. Acceptability of the trial was explored through interviews and safety was evaluated via unplanned healthcare utilisation and number of falls. Sitting, standing, stepping and sarcopenia were measured to evaluate potential intervention effects.

**Results:**

Sixty participants were recruited. Recruitment and retention rates were 72% and 83%, respectively. Completion rates for outcome measures ranged from 70 to 100%. The trial was safe (< 1 fall per participant on average at each timepoint) and trial procedures were acceptable. Descriptive analysis (mean ± SD) showed that daily sitting was 25.1 ± 82.1 min/day lower in the intervention group, and 6.4 ± 60.5 min/day higher in the control group, at 6 months compared with baseline. Hand grip strength and sit-to-stand score were improved by 1.3 ± 2.4 kg and 0.7 ± 1.0, respectively, in the intervention group.

**Conclusions:**

This study demonstrates the feasibility and safety of delivering and evaluating a remote intervention to reduce and break up sitting in older adults with frailty. The intervention showed evidence towards reducing daily sitting and improving sarcopenia, supporting its evaluation in a definitive randomised controlled trial.

**Trial registration:**

ISRCTN registry (registration number: ISRCTN17158017). Registered 6th August 2021.

**Supplementary Information:**

The online version contains supplementary material available at 10.1186/s12877-024-05310-9.

## Background

Sarcopenia can be defined as the age-related progressive and generalised loss of muscle mass and function [1]. The prevalence of this condition was 4.6% and 7.9% of UK community-dwelling older males and females, respectively [2], but could be present in up to 35% of older adults [3]. Sarcopenia is associated with an increased risk of functional disability and dependence in activities of daily living (ADL) [4, 5]. Furthermore, the risk of falls, cardiovascular disease, unplanned hospital admissions, early death and quality of life are all unfavourably associated with sarcopenia [5]. Sarcopenia is a key biological driver in the development of frailty [6], which can be defined as a syndrome characterised by reduced reserve and resistance to stressors [7]. The increased vulnerability associated with diminished strength and physiological function in frailty is also mediated by sarcopenia [6]. As individuals with mild frailty have a significantly increased risk of nursing home admission, unplanned hospitalisation and mortality (89%, 93% and 92% risk, respectively, over a 1-year period), which rises substantially with moderate and severe frailty [8], effective management to limit the progression of sarcopenia is crucial.

Older adults in the general population engage in sedentary behaviour for an average of 9.5 h per day [9]. Increasing volumes of sedentary time are detrimentally associated with physical function, muscle mass and sarcopenia [10–12]. As sarcopenia is a biological substrate for frailty, it is plausible that sedentary time is also related to this syndrome. This is supported by evidence that each additional hour of sedentary time is associated with an increased odds of frailty [13]. Moreover, community-dwelling individuals with pre-frailty and frailty spend 86 and 73 min more per day, respectively, being sedentary than their non-frail counterparts [14]. Reducing sedentary time may, therefore, represent an intervention target for the management of sarcopenia in older adults with frailty.

In addition to reducing daily sedentary time, increasing the number of breaks in sedentary time and limiting time spent in prolonged sedentary bouts may need to be specifically targeted for the management of sarcopenia and frailty. A lower number of breaks in sedentary time was associated with higher odds for impairment in ADL in older adults [15]. On the contrary, increased breaks in sedentary time are associated with reduced odds of pre-sarcopenia [11] and improved physical function, independent of total sedentary time [16]. Reducing and breaking up sedentary time may initially be a more feasible and acceptable strategy than moderate-to-vigorous physical activity (MVPA) for older adults with physical impairments and morbidity [17], especially in relation to overcoming barriers around risk of injury, pain and fatigue [18, 19]. A focus on reducing and breaking up sedentary time is further supported given that the adverse associations of these behaviours with sarcopenia and frailty-related outcomes appear to be independent from MVPA [12, 13, 15, 16].

Individually based behaviour change interventions to reduce sedentary time are effective in community-dwelling older adults [20]. A combination of behaviour change techniques (BCTs) have been used in these interventions, such as providing information, goal setting, self-monitoring and feedback on behaviour [20]. It is unclear if interventions in the general older adult population are generalisable to individuals with frailty who are more sedentary and face unique barriers related to diminished physical function and difficulty with ADL [6, 14]. An intervention targeting increases in standing exercises in older adults with frailty, supported by health education and telephone consultations, led to a 30 ± 10 min per day reduction in sedentary time after 16 weeks [21]. This study did not assess health and wellbeing outcomes, which limits conclusions regarding clinical effectiveness [21]. A pilot study in a small sample of older adults with frailty (*n* = 23) found improvements in the number of breaks in sedentary time, physical function and quality of life in response to a 14-week intervention comprising motivational interviewing sessions, feedback on physical function and a wearable device that provided real-time feedback and prompts to break up sitting [22]. However, the intervention did not change daily sedentary time, the effects were not compared to a usual care control group and there was high participant attrition (45%) [22]. A study that addresses the limitations of small-scale studies, short intervention periods, non-randomised controlled trial designs and assesses sarcopenia-related outcomes is warranted.

Interventions that are delivered remotely may be especially relevant for community-dwelling older adults with frailty who have difficulty with ADL, such as going outside of the home and travelling independently; factors that are related to increased sedentary time in older adults [23]. Remote delivery may also offer the opportunity for individuals to engage who are physically or socially isolated, are physically distancing to avoid infectious disease or live in rural areas. The feasibility and safety of delivering and evaluating a remote intervention to reduce and break up sedentary time in older adults with frailty should, therefore, be investigated.

The aim of this study was to conduct a randomised controlled feasibility trial of a remotely delivered sedentary behaviour intervention to improve sarcopenia and independent living in older adults with frailty. The primary objectives of the study that are reported here were to:

1. Establish and refine a strategy for recruiting older adults with frailty.
2. Determine attrition rates of participants in the trial.
3. Determine data completion rates for the study outcome measures.
4. Assess reasons for taking part in the study.
5. Assess acceptability and experiences of randomisation to usual care and completing the study measurements.
6. Assess safety of the trial.

The secondary objective was to explore the intervention’s potential efficacy for improving sitting, standing and stepping, sarcopenia, physical function, sarcopenia-related quality of life, mood and wellbeing.

## Methods

### Study overview

This was a two-arm mixed-methods randomised controlled feasibility trial, reported following Consolidation Standards of Reporting Trials guidelines for pilot and feasibility trials [24]. The study protocol was published in full [25] and the research was conducted in accordance with the Declaration of Helsinki. Following baseline assessments, participants were individually randomised to the Frail-LESS (LEss Sitting and Sarcopenia in Frail older adults) intervention or usual care control group for six months. Study assessments were then repeated at 3- and 6-months following allocation to the study arms. Participants and researchers were blinded to group allocation until baseline assessments were completed.

### Study setting

The study took place in the London metropolitan area, England. Measurements took place at Brunel University London or the home of participants according to their ability to travel and personal preference.

### Recruitment

Participants were recruited from General Practitioner (GP) practices located in the North West of London, in addition to a clinical research recruitment organisation (Lindus Health) using paid Facebook advertising. Individuals were also recruited from the Brunel Older People’s Reference Group (via email) and word of mouth. Individuals who expressed interest in the study were contacted by a researcher to undergo screening by telephone and email.

### Eligibility criteria

Eligible participants were community-dwelling adults aged at least 65 years and defined as having very mild or mild frailty using the Clinical Frailty Scale version 2.0 [26]. According to this scale, individuals with very mild frailty have symptoms that limit activities but are not dependent on others, while individuals with mild frailty have more evident symptoms and are dependent on others for high order instrumental activities of daily living e.g. heavy housework. Participants were eligible if they sat for at least 60% of their waking day (measured using the International Physical Activity Questionnaire [27]) and were able to ambulate independently (with or without a walking aid) on a level surface i.e. a Functional Ambulation Category rating of ≥ 4 [28].

Individuals were excluded from the study if they were unable to stand or walk due to a physical or mental impairment, had a score ≥ 7 in the Six-Item Cognitive Impairment Test [29] or were unable to communicate in English to enable them to engage fully in the study.

### Sample size

It is recommended that sample sizes for feasibility studies are not informed by power calculations [30, 31]. Instead, pilot and feasibility study sample sizes should be based on being sufficient to address the study aims and generating a SD for the primary outcome to inform the sample size in a definitive randomised controlled trial (RCT) [30, 31]. This study aimed to recruit 60 participants with the intention that at least 40 would be retained at 6 months. In line with pilot and feasibility study guidelines [30, 31], this was considered to be appropriate for generating a SD and determining the feasibility, acceptability and safety of the trial and intervention.

### Randomisation

Participants were randomised individually to the intervention or usual care control group on a 1:1 ratio using a fixed block size of four. Randomisation was concealed and undertaken by an independent researcher using www.randomizer.org. Participants and researchers were unblinded to group allocation following randomisation due to the nature of the study.

### The Frail‑LESS intervention

The Frail-LESS (LEss Sitting and Sarcopenia in Frail older adults) multicomponent intervention was developed using the Behaviour Change Wheel framework, drawing from a COM-B (Capability, Opportunity, Motivation-Behaviour) analysis [32] and using the BCT Taxonomy for intervention content [33]. The intervention was delivered remotely and included use of technology and individual tailoring, which were identified as being appropriate to support reducing and breaking up sitting [25]. The full intervention development process was described previously [25]. At the start of the intervention, participants were sent a psychoeducation workbook, tailored feedback and a wearable device. Health coaching and peer support sessions were offered thereafter.

#### Psychoeducation workbook

A psychoeducation workbook was provided in hard copy format (Additional file 1), which was based on previous interventions that effectively reduced sitting [34, 35]. The workbook included information tailored to older adults regarding the health risks associated with sitting too much, the potential therapeutic effects of reducing and breaking up sitting, aims and ideas for limiting sitting, goal setting, action planning and problem solving.

#### Tailored feedback

A personalised feedback sheet on daily sitting, standing, stepping and breaks in sitting was provided to each participant at baseline, 3- and 6-months using data collected at each respective timepoint (see example in Additional file 2).

#### Wearable device

Each participant was provided with a wrist-worn Garmin Vivofit 4 device (Garmin Ltd. Kansas, U.S.) and guidance for downloading the accompanying app to use throughout the intervention. This device provides feedback on inactive time via a coloured ‘move bar’ that partially fills across the top of the display if the participant has been inactive (< 100 steps) for 60 min, with a further two segments of the bar filling with each additional 15 min of inactivity. This is accompanied by an audible alert each time the bar fills to prompt the participant to ambulate for a few minutes (equivalent to ~ 200 steps) to reset the bar. The Vivofit also provides feedback on steps and energy expenditure, and has a daily step goal function.

#### Health coaching

Tailored one-to-one support was provided using a previously developed consultation style [35] that draws from motivational interviewing (MI) [36] and health coaching using the GROW (goal, reality, options, will/way forward) model [37]. The consultations included a COM-B real-time analysis of the barriers to breaking up and reducing sitting, and the delivery of tailored BCTs. The individuals providing the health coaching had backgrounds in psychology and behaviour change and received training by A.M.C on how to use this consultation style specifically for this study population. Training consisted of the core principles for MI, health coaching and using the COM-B model, alongside the BCTs that are included in the intervention and ways that they can be delivered. Initial training was over a 3-hour period, with booster sessions to check knowledge and for skill development. Health coaching sessions took place within five days after the intervention started with further sessions at approximately 2, 6, 12 and 18 weeks. The sessions took place over video call or telephone to suit the preference of each participant.

#### Peer support

A virtual peer support group was set up by the research team that participants had the option to join. Monthly meetings took place over Microsoft Teams and centred on participants discussing their experiences with the intervention, barriers and problem solving, trying out different activities to support less sitting and participants providing each other with general support. A WhatsApp group was created by one of the participants for interaction outside of the support meetings.

### Control group

This group continued any usual healthcare as normal and did not receive any additional intervention during the study. At the end of the study, participants in this group had the option of a tailored feedback sheet based on their 6-month activPAL measurements, the psychoeducation workbook and a Garmin Vivofit.

### Study measurements and outcomes

#### Trial feasibility and safety

Trial feasibility was assessed in terms of participant eligibility, recruitment, retention and data completion rates for the planned primary outcome (sarcopenia) in a definitive trial. Safety of the trial was assessed in terms of unplanned hospital and GP visits, the number of falls at each timepoint, and harms or unintended side effects as a result of participation in the study. Pain and fatigue were measured at each timepoint to evaluate intervention safety using a 100-mm visual analogue scale [38] and the Fatigue Severity Scale (Cronbach α = 0.93) [39], respectively.

#### Acceptability of the trial

Semi-structured interviews were conducted with a subset of participants from each group (*n* = 27) to explore acceptability of the measurement procedures and the potential behavioural impact of having these measurements taken. A process evaluation questionnaire included rating scale questions on the behavioural impact of completing the study measurements. The interviews also explored acceptability of participants being randomised to the control group. The interview guide and process evaluation questionnaire were informed by previous research [40].

#### Demographics

Demographic information was collected at baseline including age, ethnicity, sex, employment status, type of dwelling and home circumstances (e.g. living alone, access to green spaces and stairs).

#### Expected outcomes for a full trial

The following outcomes were measured at baseline, 3 months and 6 months. The procedures for each measurement are described in full previously [25].

##### Sarcopenia and physical function

The planned primary outcome for a definitive trial was objectively measured sarcopenia. This was assessed following the European Working Group on Sarcopenia in Older People guidelines [41]. Muscle mass was estimated by bioelectrical impedance using the Bodystat 1500 (Bodystat Ltd, Isle of Man). A hand grip dynamometer was used to measure hand grip strength whilst standing (Takei Scientific Instruments Co., Ltd, Niigata, Japan). The Short Physical Performance Battery (SPPB) was used to measure normal walking speed, standing balance and rising from a chair [42]. Participants also completed the SARC-F (Strength, assistance with walking, rising from a chair, climbing stairs, and falls) questionnaire [43], which has moderate-to-good test-retest reliability (intra-class correlation coefficient [ICC] ≥ 0.5 to < 0.9) [44]. Self-reported difficulty with ADL was assessed using the Groningen Activity Restriction Scale (Cronbach’s α = 0.89 and 0.92 for males and females, respectively) [45].

##### Sitting, standing and stepping

An activPAL4 (PAL Technologies, Glasgow, Scotland) was worn continuously for seven consecutive days on the anterior of the right thigh to measure sitting, standing and stepping outcomes. These outcomes included daily sitting, standing and stepping time; number and time in sitting bouts lasting < 30, ≥ 30 and ≥ 60 min; number and time in standing bouts lasting < 30 and ≥ 30 min; and number of sit-to-upright transitions. Participants completed a daily diary to record sleep and wake times, and any times the activPAL was removed. Data was downloaded using PAL Batch (PAL Technologies, Glasgow, Scotland) and then processed and summarised using Processing PAL V1.41–21,022,022 (University of Leicester, UK) to identify valid waking wear data [46]. Criteria for a valid day was ≥ 10 h of waking wear time, < 95% of time spent in a single event (i.e. sitting, standing or stepping) and ≥ 250 steps.

##### Height, weight and body fat

Height and weight were measured using a portable stadiometer (Seca 213; Seca GmbH, Hamburg, Germany) and electronic weighing scales (Seca 875; Seca GmbH, Hamburg, Germany), respectively. The Bodystat 1500 provided body fat % and fat mass data.

##### Mood, wellbeing, quality of life and health service use

The Sarcopenia Quality of Life (SarQol) questionnaire was used to assess sarcopenia-specific quality of life across physical and mental health, locomotion, body composition, functionality, ADL, leisure activities and fears domains; this questionnaire has excellent test-retest agreement with an ICC of 0.91 [47]. Mood was assessed using the 20-item Positive and Negative Affect Schedule [48]; Cronbach’s α = 0.89 for the positive affect scale and 0.85 for the negative affect scale [49]. Subjective wellbeing (life satisfaction, worthwhileness, happiness, anxiety) was assessed with the Office for National Statistics 4-item scale [50]. Participants self-reported their use of health services and prescribed medication using an adapted version of the Client Service Receipt Inventory [51].

### Data analysis

Trial feasibility was assessed in relation to eligibility (eligible individuals / individuals assessed for eligibility x 100), recruitment (individuals randomised / individuals who were eligible x 100) and retention rates (participants with measurements at each timepoint / participants enrolled into study x 100). Data completion rates were calculated as the number of datasets for each outcome measure / number of participants enrolled into the study x 100. The number of falls and ratings of pain and fatigue at each timepoint were analysed descriptively to assess trial safety, in addition to the number of unplanned hospital and GP visits during the study.

Interviews were transcribed verbatim and analysed using the Framework Method [52]. Coding was undertaken by L.J.M to identify themes regarding participants’ reasons for taking part in the study, experiences of randomisation to the usual care control group, and acceptability of data collection procedures. Transcripts were coded deductively to these research questions, with further exploration taking place within each theme. Process evaluation questionnaire data is reported descriptively and presented as *n* and %.

Descriptive statistics (mean ± SD) were used to explore the potential efficacy of the intervention for improving the intended primary (sarcopenia) and secondary outcomes in a definitive trial. Microsoft Excel v16.0 (Microsoft Corporation, Redmond, Washington, USA) and SPSS v26.0 (IBM Corp., Armonk, NY, USA) were used to conduct this analysis. Significance testing was not undertaken as formal sample size calculations for the trial were not used, in line with pilot and feasibility study guidelines [30, 31] and the study protocol [25].

### Progression criteria to a definitive trial

The following criteria were used to judge progression to a definitive trial:

- Recruitment of the target sample size within the intended timeframe.
- Valid primary outcome data for ≥ 70% of study participants.
- The intervention was delivered as planned.
- The trial and intervention were safe and acceptable to participants.

## Results

### Trial feasibility

Recruitment and eligibility information is presented in Additional file 3. Fifty-two GP practices agreed to support participant recruitment for the study and sent out text messages to 11,910 potentially eligible patients. Of these, there were 239 patients who expressed an interest in taking part. Further expressions of interest were received via Lindus Health (*n* = 66), Brunel Older People’s Reference Group (*n* = 3) and word of mouth (*n* = 3). The eligibility rate of the 198 individuals screened was 42%, with 72% (*n* = 60) of these being recruited into the study. Baseline characteristics of the participants are shown in Table 1.

**Table 1:** Baseline characteristics of the sample

		Intervention group (*n* = 30)		Control group (*n* = 30)		Whole sample (*n* = 60)	
		*n*	%	*n*	%	*n*	%
Age (years), (mean ± SD)		75 ± 7		74 ± 6		74 ± 6	
Sex	Female	20	67%	20	67%	40	67
	Male	10	33%	10	33%	20	33
Ethnicity	Asian or Asian British	2	7%	3	10%	5	8
	Black, African, Caribbean, or Black British	2	7%	1	3%	3	5
	Other	1	3%	1	3%	2	3
	White	25	83%	25	83%	50	83
Employment Status	Disabled and unable to work	1	3%	0	0%	1	2
	Employed full-time	1	3%	0	0%	1	2
	Employed part-time	3	10%	4	13%	7	12
	Retired		0%	25	83%	50	83
	Unemployed looking for work	25	83%	1	3%	1	2
Type of Dwelling	Bungalow	2	7%	2	7%	4	7
	Flat	15	50%	6	20%	21	35
	House	12	40%	22	73%	34	57
	Sheltered Accommodation	1	3%	0	0%	1	2
Home Circumstances^a^	Live alone	18	60%	12	40%	30	50
	Have access to green space	28	93%	27	90%	55	92
	Have stairs inside home	17	57%	22	73%	39	65
	Have access to stairs at home	11	37%	12	40%	23	38

^a^% calculated as number of participants who answered ‘Yes’ to each question / N of group or sample

**Fig. 1:**
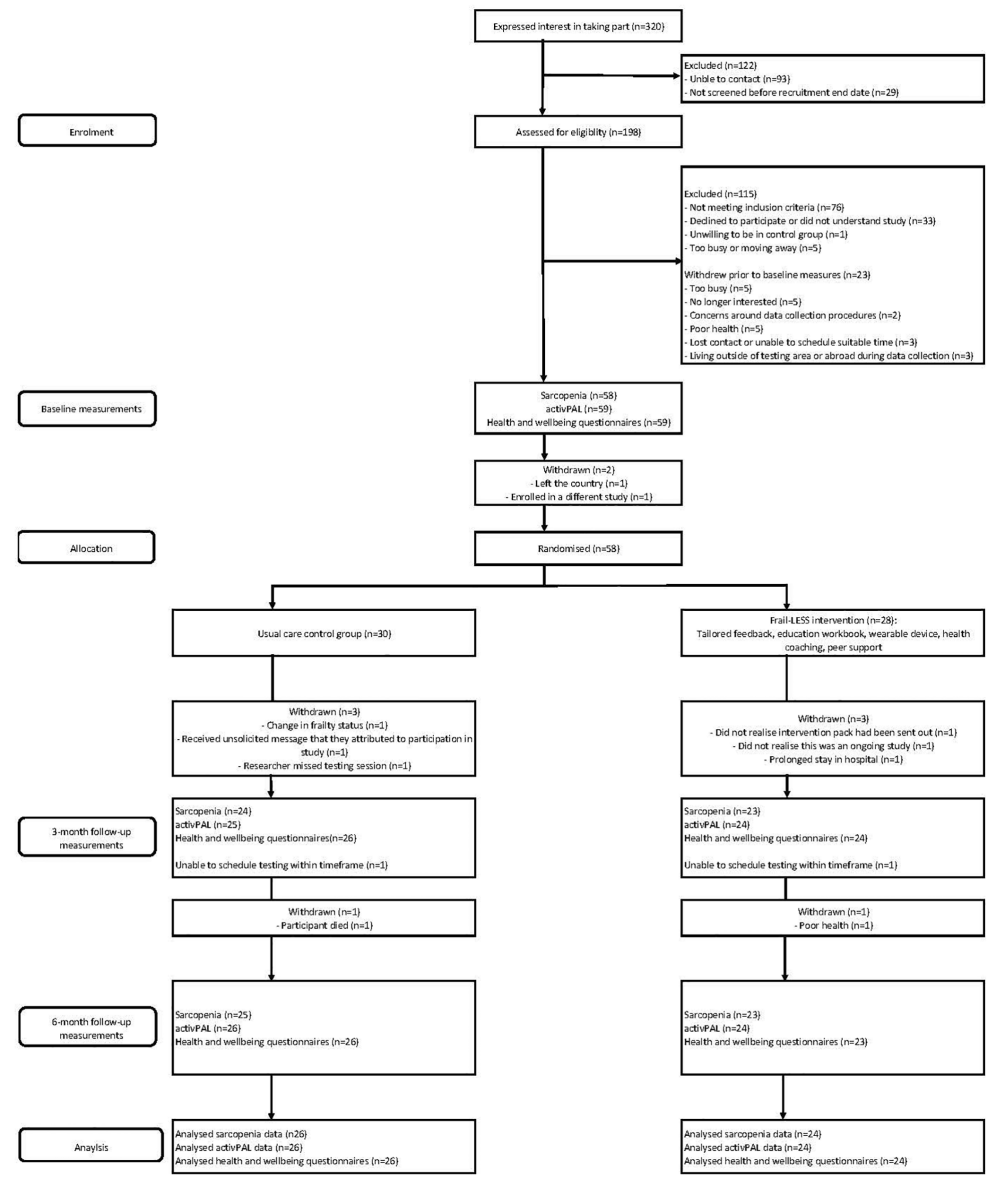
Study flow diagram

Figure 1 shows participant flow during the study. The recruitment period was November 2021 to June 2022, with baseline data collection taking place during this same period. The 3- and 6-month measurements occurred February to September 2022 and May to December 2022, respectively. Eight participants withdrew at the 3-month timepoint (control *n* = 3; intervention *n* = 5) and a further two withdrawals (*n* = 1 for each group) at the 6-month timepoint; this gave an overall retention rate of 83% (Additional file 3). Data completion rates for the expected primary outcome (sarcopenia) in both groups was 97% (*n* = 29) for muscle mass and 100% (*n* = 30) for both hand grip strength and the SPPB at baseline. At 3- and 6-months, this ranged from 77 to 87% (Table 2). For the activPAL measurement, data completion rates were 100% and 97% for the control and intervention participants, respectively, at baseline. Completion rates for the activPAL ranged from 80 to 93% at 3- and 6-months. Health and wellbeing questionnaires and the Client Service Receipt Inventory were completed by 100% and 97% of the control and intervention participants, respectively, at baseline, and ranged from 73 to 87% at the 3- and 6-month timepoints.

**Table 2:** Completion rates for the study measurements at each timepoint. Data shown as n (%)

	Baseline		3 months		6 months		All timepoints	
	Control	Intervention	Control	Intervention	Control	Intervention	Control	Intervention
activPAL data								
≥ 1 valid day	30 (100)	29 (97)	25 (93)	24 (80)	26 (87)	24 (80)	24 (80)	24 (80)
≥ 2 valid days	30 (100)	29 (97)	23 (85)	24 (80)	26 (87)	24 (80)	22 (73)	24 (80)
≥ 3 valid days	30 (100)	29 (97)	23 (85)	24 (80)	24 (80)	24 (80)	22 (73)	24 (80)
≥ 4 valid days	30 (100)	29 (97)	23 (85)	24 (80)	24 (80)	24 (80)	22 (73)	24 (80)
Sarcopenia								
Muscle mass	29 (97)	29 (97)	24 (80)	23 (85)	25 (83)	23 (77)	23 (77)	21 (70)
Hand grip strength	30 (100)	30 (100)	24 (80)	24 (80)	25 (83)	23 (77)	23 (77)	21 (70)
SPPB	30 (100)	30 (100)	25 (83)	24 (80)	26 (87)	23 (77)	24 (80)	21 (70)
Health and wellbeing questionnaires	30 (100)	29 (97)	26 (87)	24 (80)	26 (87)	23 (77)	25 (83)	22 (73)
Client Service Receipt Inventory	30 (100)	29 (97)	26 (87)	24 (80)	26 (87)	23 (77)	25 (83)	22 (73)

Data completion rates were calculated as the number of complete datasets for each outcome measure / number of participants enrolled into the study x 100. SBBP, Short Physical Performance Battery

Health and wellbeing questionnaires included the following: SARC-F, Groningen Activity Restriction Scale, SarQol, Positive and Negative Affect Schedule, and Subjective wellbeing

### Trial safety

No harms or unintended side effects occurred during the study. The number of falls during the previous 3 months was < 1 on average per participant at each timepoint, with a similar number for each the control and intervention groups (Additional file 3). Similar frequencies were seen for the number of unplanned hospital and GP visits in both groups at each timepoint (< 1 visit per participant on average) (Additional file 3). Pain ratings in the control group increased by 5.2 ± 22.7 and 1.8 ± 25.0 at 3 and 6 months, respectively, compared with baseline (Additional file 3). In the intervention group, pain was 2.9 ± 31.1 higher at 3 months, but at 6 months was 5.4 ± 29.3 lower compared with baseline. Fatigue in the control group decreased by 0.3 ± 1.4 and 0.2 ± 1.2 at 3 and 6 months, respectively, compared with baseline (Additional file 3). For the intervention group, fatigue decreased by 0.5 ± 1.4 and 0.5 ± 1.5 at 3 and 6 months, respectively.

### Acceptability of the trial

Interviews with 13 control and 14 intervention participants identified the following themes in relation to acceptability of the trial.

#### Reasons for taking part in the study

Many participants stated that their initial decision to participate in the study was influenced by their physical ailments, including poor mobility, falls, high cholesterol, and arthritis. These participants appeared to be aware of their sedentary behaviour and hoped that involvement with the Frail-LESS intervention could help them regain mobility and become more active again.

*“[I had] a lot of aches and pains*,* and I just wasn’t doing anything. Just sitting and watching television for long periods of time.”* (FL32 [participant ID], Intervention)

*“[…] the idea that I would do more exercise*,* be encouraged to move around more. That’s what motivated me*,* because it’s too tempting to just do not too much.”* (FL02, Intervention).

Many participants expressed a desire to help others and support research by participating.

*“I have been under the National Health for years*,* they’ve kept me alive and I wanted to give something back.”* (FL28, Control).

*“I’m always happy to help out with research that might help other people.”* (FL11, Intervention).

The timing of the study played a key role in participants’ decisions to participate. In particular, having available time due to retirement and recruitment occurring during the COVID-19 pandemic (when self-isolation restrictions following a positive COVID-19 test were in place), appeared to motivate participants to take part.

*“Basically*,* I’m retired. So*,* I thought*,* Well*,* why not join in and see what it’s all about?”* (FL52, Intervention).

*“It was COVID - Not doing anything so might as well.”* (FL37, Control).

Most participants heard about the study through their GP, therefore recommendation from a credible source may have influenced their decision to take part.

*“My doctor recommended it to me. So that’s the reason I did it.”* (FL20, Control).

#### Acceptability of randomisation to the control group

Though some control participants acknowledged it would have been nice to be part of the intervention group, none were upset when informed of their group allocation and understood the importance of their contribution to the study.

*“I think I would have liked to have been in the other group. But it didn’t matter. The fact I was in the control group*,* I understand that you’ve got to have people to compare to.”* (FL13, Control).

*“We’re the control group. So*,* no*,* I wasn’t expecting anything to change for me. It was just that they were collecting data. And that’s what I was contributing to.”* (FL50, Control).

#### Acceptability of study measurements

All participants described the study measurements as acceptable and non-intrusive.

*“That [the measurements] was totally unintrusive. It was fine. It was all very easy indeed.”* (FL28, Control).

Participants were positive about the friendliness and professionalism of the study personnel who conducted the measurement sessions.

*“He [the researcher] was very courteous*,* very professional […] He explained everything very well. And it was all done very*,* very easily.”* (FL06, Intervention).

A small number of participants commented on their surprise at finding their height had reduced.

*“I found that I was about two inches shorter than I used to be […] And it didn’t bother me. But it might do some people […] because I think some people are a lot more sensitive about it.”* (FL13, Control).

One participant commented that having measurements taken at home, rather than at a University campus, meant that they felt more comfortable and performed better.

*“Probably more relaxed here [in own home]. One-on-one in me own home. So yeah*,* probably more relaxed*,* because obviously the doors were shut. He [the researcher] put a little marker on the floor. I seemed to do alright. And*,* um*,* obviously*,* you’re walking on your own floor as well.”* (FL35, Intervention).

A few participants noted the cold temperature of the University room in which the measurements were taken, and some had issues accessing the building.

*“I have to say it was damn cold in there [University building]*.” FL52, Control)

*The first time I went to [University campus] it took me 2 and a half hours to get there and it was freezing cold […] But when he [the researchers] offered to do a home visit*,* I said yes please […] I think if I had had to go to [University campus] for the second session*,* I would have dropped out.”* FL10, Intervention]

#### Behavioural changes due to measurements and general involvement in the study

For participants in the intervention group, many felt that having measurements taken had not impacted on their behaviour.

*“As I said*,* I don’t know what the readings were. So*,* it just didn’t influence me in any way.”* (FL32, Intervention).

*“No impact whatsoever [on behaviour].”* (FL52, Intervention).

However, some intervention participants commented that the measurements encouraged them to adhere to reducing sedentary behaviour.

*“I think it [the measurements] made me adhere to the study*,* knowing that things were being measured […] It’s certainly a reminder that you need to do the things that you’ve signed up for.”* (FL06, Intervention).

Some control participants acknowledged the influence of the study measurements (and general involvement with the study) on their behaviour, whereas others felt there had been no change as a result of taking part.

*“The honest truth is it [the study] didn’t impact me at all. None of it. I didn’t change anything.”* (FL28, Control).

*“I’ve genuinely been conscious over recent months and thinking yes*,* you are moving better.”* (FL25, Control).

One participant was conscious of whether they should be making such changes as they were in the control group:

*“It [having measurements taken] made quite a bit of difference […] I started practicing the balance more. I started standing*,* exercising*,* standing up and sitting down quickly. And then I thought to myself*,* I’m in the control group […] Is it right to make a difference in my life when I’m the control?”* (FL46, Control).

In fact, in the process evaluation questionnaire, 35% of control participants at 3 months believed they had changed their sitting behaviour as a result of taking part in the study, with that number rising to 46% at 6 months (Additional file 4). Intervention participants strongly agreed or agreed (88% and 83% at 3 and 6 months, respectively) that the measurement sessions motivated them to change their sitting time, compared with control participants (54% and 58% at 3 and 6 months, respectively).

### Potential of the intervention to improve Sarcopenia

Descriptively, hand grip strength reduced by 0.7 ± 1.4 and 0.6 ± 1.3 kg in the control and intervention groups, respectively, at 3 months compared with baseline (Table 3). At 6 months, the intervention group’s grip strength increased by 1.3 ± 2.4 kg, with the control group’s being relatively unchanged (increasing by 0.1 ± 3.8 kg). The intervention group’s sit-to-stand score increased by 0.6 ± 0.6 and 0.7 ± 1.0 at 3 and 6 months, respectively; for the control group, the score reduced by 0.2 ± 0.8 and increased by 0.2 ± 1.0 at these respective timepoints. Changes in percent muscle mass appeared to be similar for the control and intervention participants with increases of 1.2 ± 3.1 and 1.5 ± 2.9%, respectively, at 6 months. Balance and normal walking speed did not appear to change. The overall SPPB score improved by 0.6 ± 1.3 and 0.9 ± 1.8 for the intervention participants, and by 0.4 ± 1.3 and 0.8 ± 1.4 for the control participants, at 3 and 6 months, respectively. As shown in Additional file 3, there were improvements in overall SarQol and each domain for both the intervention and control participants at 3 and 6 months. Overall SarQol was 6.0 ± 8.2 and 6.5 ± 13.1 higher at 6 months in the control and intervention participants, respectively, compared with baseline.

**Table 3:** Descriptive statistics for body composition and sarcopenia. Data presented as mean and SD

	Baseline		3 Months		6 Months		Change (baseline to 3 months)		Change (baseline to 6 months)	
	Control	Intervention	Control	Intervention	Control	Intervention	Control	Intervention	Control	Intervention
	(*n* = 30)	(*n* = 30)	(*n* = 25)	(*n* = 24)	(*n* = 26)	(*n* = 23)	(*n* = 25)	(*n* = 24)	(*n* = 26)	(*n* = 23)
Fat mass (%)	40.7	41.7	41.2	43.1	39.5	41.5	0.4	-0.3	-1.2	-1.5
	8.4	9.8	8.9	8.9	8.4	9.2	3.9	2.6	3.1	2.9
Fat mass (kg)	34.0	36.0	34.0	37.9	32.7	36.4	-0.5	-0.6	-1.8	-1.2
	9.3	14.1	9.4	14.4	9.2	15.6	3.3	3.0	3.9	4.2
Lean mass (%)	59.3	58.3	58.8	56.9	60.5	58.5	-0.4	0.3	1.2	1.5
	8.4	9.8	8.9	8.9	8.4	9.2	3.9	2.6	3.1	2.9
Lean mass (kg)	49.6	49.0	48.5	48.7	50.0	49.5	-1.4	-0.6	-0.2	1.0
	12.2	12.2	11.6	12.1	11.1	12.4	4.3	3.1	2.9	2.2
Body mass index (kg/m^2^)	31.0	31.7	30.6	32.3	30.4	32.1	-0.7	-0.5	-0.9	-0.1
	4.6	6.7	4.4	7.0	4.4	7.4	1.4	1.3	1.5	1.6
Hand Grip Strength (kg)	24.5	25.0	24.1	24.3	25.1	25.9	-0.7	-0.6	0.1	1.3
	7.0	7.3	6.4	6.5	6.9	6.6	3.6	4.8	3.8	2.4
SPPB Balance	3.5	3.3	3.8	3.5	3.9	3.5	0.4	0.0	0.3	0.0
	0.7	1.0	0.4	0.9	0.3	0.9	0.6	0.9	0.8	1.1
SPPB sit-to-stand	2.1	1.5	2.1	2.1	2.5	2.3	-0.2	0.6	0.2	0.7
	1.2	1.1	1.3	1.3	1.1	1.1	0.8	0.6	1.0	1.0
SPPB Walking speed	3.2	3.2	3.5	3.2	3.6	3.4	0.2	0.0	0.3	0.1
	1.0	0.8	0.9	0.9	0.8	0.8	0.7	0.8	0.6	0.5
SPPB Total	8.8	8.0	9.4	8.8	9.9	9.1	0.4	0.6	0.8	0.9
	2.2	2.3	2.0	2.1	1.8	2.3	1.3	1.3	1.4	1.8

Change statistics were calculated only for participants who provided data at every time point

*n* − 1 for control fat and lean mass measures at baseline, 3 months and 6 months

*n* − 1 for intervention fat and lean mass measures at baseline and 3 months

*n* − 1 for control hand grip measures at 3 months and 6 months

Higher scores for each of the SPPB outcomes indicate better performance

SPPB, Short Physical Performance Battery

### Potential of the intervention to reduce and break up sitting

Daily sitting was 15.5 ± 88.1 and 25 ± 82.1 min/day lower at 3 and 6 months, respectively, in the intervention group compared with baseline (Table 4). For the control group, sitting was 6.9 ± 79.0 min/day lower at 3 months and 6.4 ± 60.5 min/day higher at 6 months. The reduced sitting time in the intervention group appeared to be replaced predominantly by short bouts of standing, reflected by 15.1 ± 80.0 and 20.5 ± 59.9 min/day spent more in standing bouts lasting 0 to 30 min at 3 and 6 months, respectively. Time in sitting bouts ≥ 60 min was relatively unchanged at 3 months, but reduced by 37.3 ± 107.1 min/day in the intervention group and increased by 12.7 ± 107.2 min/day in the control group, at 6 months. The number of sit-upright transitions did not appear to change in the control group and decreased by 2.4 ± 11.1 and 1.7 ± 9.0 per day at 3 and 6 months, respectively, in the intervention participants.

**Table 4:** Descriptive statistics for the activPAL variables data normalised to a 16-hour day

	Baseline		3 Months		6 Months		Change (baseline to 3 months)		Change (baseline to 6 months)	
	Control	Intervention	Control	Intervention	Control	Intervention	Control	Intervention	Control	Intervention
	(*n* = 30)	(*n* = 29)	(*n* = 25)	(*n* = 24)	(*n* = 26)	(*n* = 24)	(*n* = 25)	(*n* = 24)	(*n* = 26)	(*n* = 24)
Wear time (minutes/day)	954.3	933.6	954.9	957.3	973.9	940.4	6.8	25.7	19.9	17.9
	75.9	74.2	82.9	87.1	73.5	43.8	71.5	65.4	65.8	53.3
Sitting time (minutes/day)	670.0	692.2	653.7	670.8	676.2	668.0	-6.9	-15.5	6.4	-25.1
	121.2	92.9	133.4	123.2	141.2	124.4	79.0	88.1	60.5	82.1
Standing time (minutes/day)	217.1	201.7	230.5	217.8	213.0	218.9	6.7	14.0	-3.0	19.4
	99.8	76.8	107.8	103.4	114.5	97.1	69.6	80.7	44.0	63.5
Stepping time (minutes/day)	72.9	66.1	75.8	71.3	70.8	73.1	0.2	1.5	-3.4	5.8
	40.2	29.5	38.0	31.8	44.0	39.5	24.3	16.0	22.1	27.2
Steps per day	5727	5044	5931	5358	5626	5652	-52	-21	-245	456
	3619	2795	3429	2848	4081	3743	2407	1516	1977	2718
Number of sit-upright transitions per day	42.1	41.7	42.3	42.1	41.5	41.2	-0.4	-2.4	-0.3	-1.7
	12.5	13.5	12.0	10.0	13.0	11.9	6.7	11.1	8.6	9.0
Time in sitting bouts ≥ 30 min (minutes/day)	440.4	472.2	428.4	448.8	453.7	444.0	0.0	-6.6	11.4	-22.2
	163.2	139.2	165.6	150.6	175.8	154.8	94.2	102	98.4	107.4
Time in sitting bouts ≥ 60 min (minutes/day)	283.1	308.2	276.4	283.9	303.8	262.6	6.0	-6.6	12.7	-37.3
	164.9	161.2	162.6	140.2	170.0	136.0	103.8	114.0	107.2	107.1
Time in standing bouts < 30 min (minutes/day)	215.3	198.6	225.8	215.5	207.8	216.6	4.2	15.1	-6.4	20.5
	98.2	74.6	101.6	102.8	107.3	95.3	65.8	80.0	39.0	59.9
Time in standing bouts ≥ 30 min (minutes/day)	1.8	3.1	4.7	2.4	5.2	2.3	2.5	-1.1	3.4	-1.1
	5.3	6.8	17.9	6.7	13.6	5.3	16.9	6.9	10.9	7.6

Data shown for participants who provided at least one valid day at each time point. Change statistics were calculated only for participants who provided data at every time point

### Potential of the intervention to improve self-reported physical function, mood and wellbeing

Self-reported physical function, mood and wellbeing data is show in Additional file 3. Activity restriction (i.e. difficulty with ADL) was lower by 0.2 ± 3.3 and 1.2 ± 2.8 in the control and intervention groups, respectively, at 3 months compared with baseline. At 6 months, activity restriction was lower by 1.4 ± 4.2 in the control group and by 2.1 ± 3.2 in the intervention group. The intervention group’s positive affect improved by 2.5 ± 5.3 and 2.8 ± 6.5 at 3 and 6 months, respectively, with no apparent change in the control participants. Life satisfaction was 0.6 ± 1.1 and 0.8 ± 1.3 higher at 3 and 6 months in the intervention group, respectively, while in the control group scores were lower by 0.7 ± 2.1 and 0.8 ± 2.1. Anxiety was lower at 3 months (-1.0 ± 2.0) and 6 months (-1.5 ± 3.1) in the intervention group, with no change at 3 months and a 1.2 ± 4.2 increase at 6 months in the control group.

## Discussion

This study provides novel findings demonstrating the feasibility and safety of delivering and evaluating a remotely delivered intervention to reduce and break up sitting in older adults living with frailty. Sufficiently high eligibility and recruitment rates demonstrated that it was feasible to achieve the target sample size. However, as only 2% of patients approached via GP practices (via text message) expressed interest in taking part in the study, the recruitment strategy may need refinement in a future definitive RCT. A multi-centre study across diverse locations is, therefore, recommended in a future trial to achieve a larger and more generalisable sample size. Involving patients in the design of text message content may also increase the likelihood of patients reading these and interacting with the study information provided [53]. The use of follow-up text message reminders and telephone calls may also prompt patients to respond [54]. These strategies warrant consideration for a future RCT.

Retention of participants at 6 months was sufficiently high (83%) for progression to a definitive RCT [25]. Participants reported being motivated to take part to improve their health and make use of their spare time. The option for data collection taking place at participants’ homes encouraged retention. In contrast, retention in a 16-week study in older adults with frailty (*n* = 43) evaluating a sedentary behaviour intervention comprising of standing exercises, health education and telephone support, had a much lower retention rate (55%) [22], despite offering home data collection visits [22]. This could be explained by participants in this previous study being in poorer health, seeing as ill-health was the most common reason for withdrawal [22]. Even though dropout due to ill-health may not be completely mitigated, it is recommended that home measurement sessions are offered where possible to make participation in research more accessible for older adults with frailty.

Data completion rates for the intended primary outcome of a definitive RCT (sarcopenia) were 97–100% at baseline, 80–85% at 3 months and 70–80% at 6 months; the lower rates at 3 and 6 months being explained by participant withdrawals. Valid activPAL data was collected from 73 to 85% of participants at 3 and 6 months, which is slightly higher than previous research (72% completion rate at 16 weeks) in older adults with frailty [21]. The present study extends knowledge by demonstrating, for the first time, the feasibility of collecting sedentary behaviour, sarcopenia and independent living outcomes, to evaluate an intervention that aims to reduce sitting in older adults with frailty. The inclusion of these ageing-related outcomes in future trials will advance the development of public health and clinical care guidelines.

The trial appeared to be safe in the context of a low average occurrence of falls and unplanned hospital and GP visits during the study. There did not appear to be any descriptive indication of these outcomes being different between groups, while subjective pain appeared to be lower at 6 months in intervention participants. This suggests that the Frail-LESS intervention itself may be safe for older adults with frailty. A previous intervention targeting increased time engaging in standing exercises (involving home-based standing exercises, telephone support and health education) also reported a low number of falls and satisfactory levels of adherence in a similar sample [21]. The intervention group’s 25 min/day reduction in daily sitting at 6 months in the present study is similar to the 30 min/day decrease in daily sedentary time following the 16-week standing intervention by Tosi et al. [21]. Participants in the current study appeared to replace their sitting predominantly with short bouts of standing. Although standing time was not measured by Tosi et al. [21], it is likely that standing was the main activity used to replace sedentary time due to the standing-focused nature of the intervention. Thus, it appears that interventions to reduce sedentary time in older adults that focus on light activity, such as standing, are safe and potentially effective for sedentary behaviour change in older adults with frailty.

The intervention appeared to have potential for improving some sarcopenia outcomes (hand grip strength and sit-to-stand), mood and wellbeing, but may have limited effects on muscle mass, balance and walking speed. Improvements in timed up and go and sit-to-stand tests were seen in older adults with frailty in response to a sedentary behaviour intervention that increased the number of breaks in sedentary time [22]. Comparisons with other sarcopenia outcomes, mood and wellbeing cannot be made as these outcomes were not measured previously [22]. The lack of change in some sarcopenia outcomes in the present intervention may reflect an insufficient intervention duration (six months) or intensity of physical activity (predominantly standing) when replacing sitting. Isotemporal substitution studies have shown that reallocating 15 min/day of sedentary time with MVPA is associated with a 15% lower risk of sarcopenia, whereas substitution with light-intensity physical activity had no association [55]. However, other research has demonstrated that replacing 10 min of sedentary time/day with light-intensity physical activity was associated with lower sarcopenia risk and increased muscle mass [56]. The present study extends knowledge by demonstrating that replacing sitting with predominantly standing over a 6-month intervention could be sufficient for improving hand grip strength, sit-to-stand, mood and wellbeing outcomes. Replacing sitting with standing could be a more achievable strategy, initially, than increasing engagement in MVPA in older adults with physical impairments [17]. Investigating the effectiveness of reducing sitting via increases in standing or light-intensity physical activity over the longer-term is, therefore, warranted in a definitive RCT to appropriately inform recommendations for managing sarcopenia in older adults with frailty.

The strengths of this study include the mixed-methods design and the 6-month study period to understand, in depth, the feasibility and safety of implementing and evaluating a sedentary behaviour intervention in older adults with frailty in a definitive RCT. Furthermore, the study demonstrated that it was feasible and safe to deliver the intervention remotely. This could have important implications for delivery of behaviour change interventions to older adults who are isolated due to factors such as inability to travel, economic constraints and access difficulties. The study also included measurement of geriatric-related outcomes that have been seldom included in previous sedentary behaviour interventions with older adults. Study limitations include the sample being predominantly White, meaning generalisability to other ethnic groups is difficult. A future study should, therefore, consider strategies to aid with recruitment of participants across different ethnic groups. Suitability of the intervention for older adults from different educational and socioeconomic backgrounds should also be investigated. The intervention was delivered by the research team, which limits understanding regarding the feasibility of its implementation in routine healthcare and community settings; this should be addressed in future research. Lastly, the study may have been biased towards older adults who were familiar or willing to develop their competency in using technology.

## Conclusions

This study demonstrates the feasibility and safety of delivering and evaluating a remote intervention to reduce and break up sitting in older adults with frailty. The intervention showed evidence towards the potential for reducing daily sitting and improving sarcopenia, physical function, mood and wellbeing. These findings should be used to inform the design of definitive RCTs to evaluate the effectiveness of the Frail-LESS and similar interventions, which could subsequently lead to advancements in public health promotion and healthcare for this population group.

## Electronic supplementary material

Below is the link to the electronic supplementary material.

Additional file 1

Additional file 2

Additional file 3

Additional file 4

## Data Availability

The datasets supporting the conclusions of this article are available in Figshare, https://doi.org/10.17633/rd.brunel.26028892.v1. The raw qualitative data (transcripts) are not publicly available due to privacy restrictions. Further detail on the qualitative data and analysis that supports the findings of this study are available upon request to the corresponding author.

## Abbreviations

ADL: activities of daily living
BCTs: behaviour change techniques
COM-B: Capability, Opportunity, Motivation-Behaviour
Frail-LESS: LEss Sitting and Sarcopenia in Frail older adults
GP: General Practitioner
MI: motivational interviewing
RCT: randomised controlled trial
SarQol: Sarcopenia Quality of Life
SPPB: Short Physical Performance Battery
