# Supplementary material for: The frail-LESS (LEss sitting and sarcopenia in frail older adults) remote intervention to improve sarcopenia and maintain independent living via reductions in sedentary behaviour: findings from a randomised controlled feasibility trial
Source: BMC Geriatr. 2024 Sep 9;24:747. doi: 10.1186/s12877-024-05310-9 (PMC11382500; doi:10.1186/s12877-024-05310-9)
Supplement: Supplementary file 1 — Additional file 1 [file 12877_2024_5310_MOESM1_ESM.pdf]

Frail-LESS

Sit less.
Move more.
Live better.

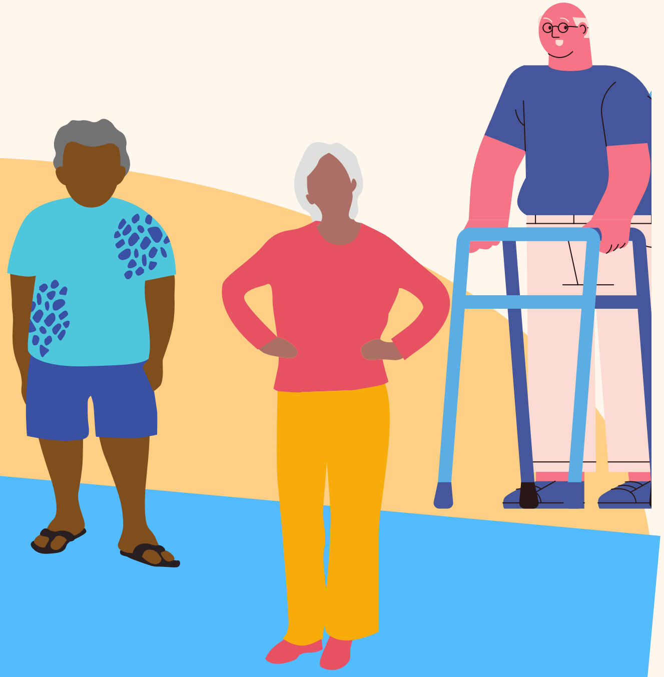

Brunel University London

University of Bedfordshire

HEALTH RISKS OF SITTING TOO MUCH

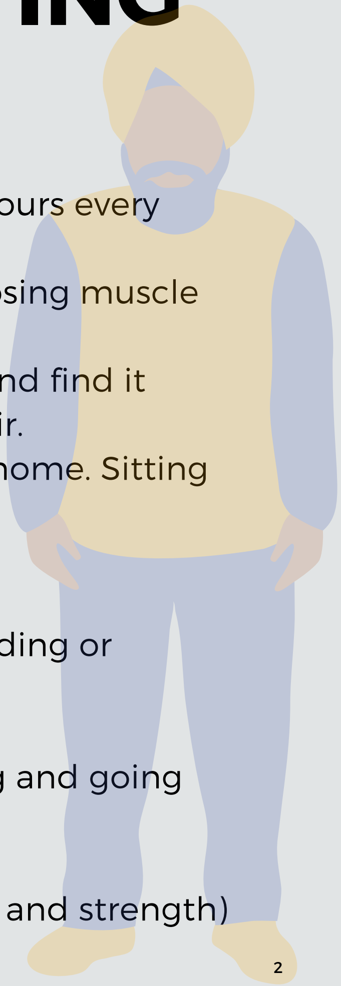

- Research shows that older adults on average sit for at least 9 hours every day.
- Every additional hour of sitting each day increases the risk of losing muscle and strength by 33%.
- Older adults who sit more have less strength, poorer balance and find it more difficult to do everyday activities like getting out of a chair.
- Mild frailty doubles the risk of going into hospital or a nursing home. Sitting less could help to reduce frailty.

The good news!

- More frequent breaks in sitting (i.e. moving from sitting to standing or walking) is related to:
  - better physical function,
  - reduced difficulty in doing everyday activities, like shopping and going up stairs,
  - lower level of frailty, and
  - 45% reduced risk of sarcopenia (which is the loss of muscle and strength) in older adults,

The Frail-LESS programme aims to support you with breaking up and reducing your sitting time, which can help you:

LOWER YOUR RISK OF LOSING MUSCLE AND STRENGTH BY 45%

FEEL GOOD AND IMPROVE YOUR MENTAL HEALTH

IMPROVE HEART HEALTH AND LOWER YOUR RISK OF A HEART ATTACK OR STROKE

CARRY OUT EVERYDAY ACTIVITIES LIKE WALKING, CLIMBING STAIRS, AND GETTING OUT OF A CHAIR

IMPROVE YOUR BALANCE

MAINTAIN YOUR INDEPENDENCE FOR LONGER

SITTING GUIDELINES

WHAT TO AIM FOR

LIMIT YOUR SITTING TIME

- Ideally, sit for less than 30 minutes at a time
- Take short (2-3 minute) frequent breaks throughout the day
- Light intensity activities, like standing up or slow walking, count as 'breaks'
- Get up at least once an hour

IDEAS FOR REDUCING YOUR SITTING TIME

- When reading, set an alarm to remind you to get up often
- Stand up while the kettle is boiling
- Stand up or walk around while on the phone
- Walk around the garden or your home when your activity tracker prompts you to get up
- Stand up during TV adverts and do some stretches

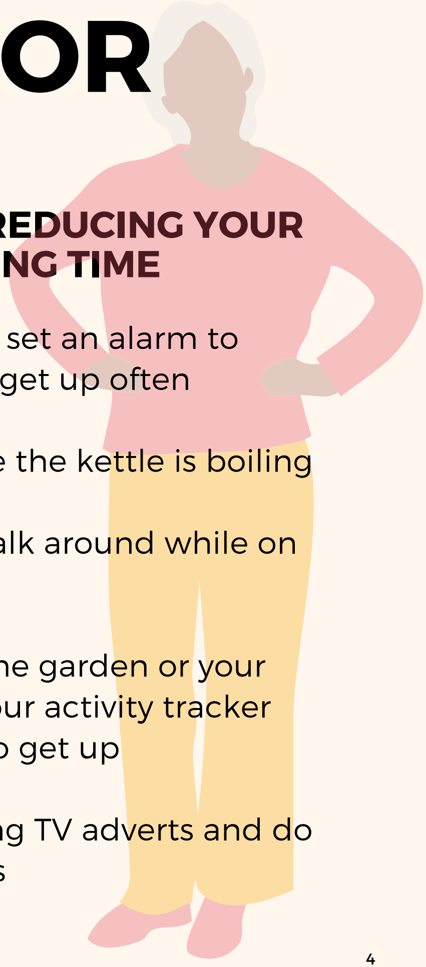

YOUR GOALS

Think about **what** you want to achieve and **how** you are going to do it. Think about the feedback you had on your sitting time and then write your personal goals below.

For example, 'I will break up my sitting every hour'.

Short term goal
(over the next 2 weeks):

How confident are you?
Not at all 1-2-3-4-5-6-7-8-9-10 Very

Long term goal
(over the next few months):

How confident are you?
Not at all 1-2-3-4-5-6-7-8-9-10 Very

How to change

STEPS FOR SUCCESS

Make a note of the things you are going to do to break up and reduce your sitting time. Break it into small steps that will add up over time.

What am I going to do?	Where am I going to do it?	When & how long am I going to do it?	Who am I going to do it with?
○			
○			
○			

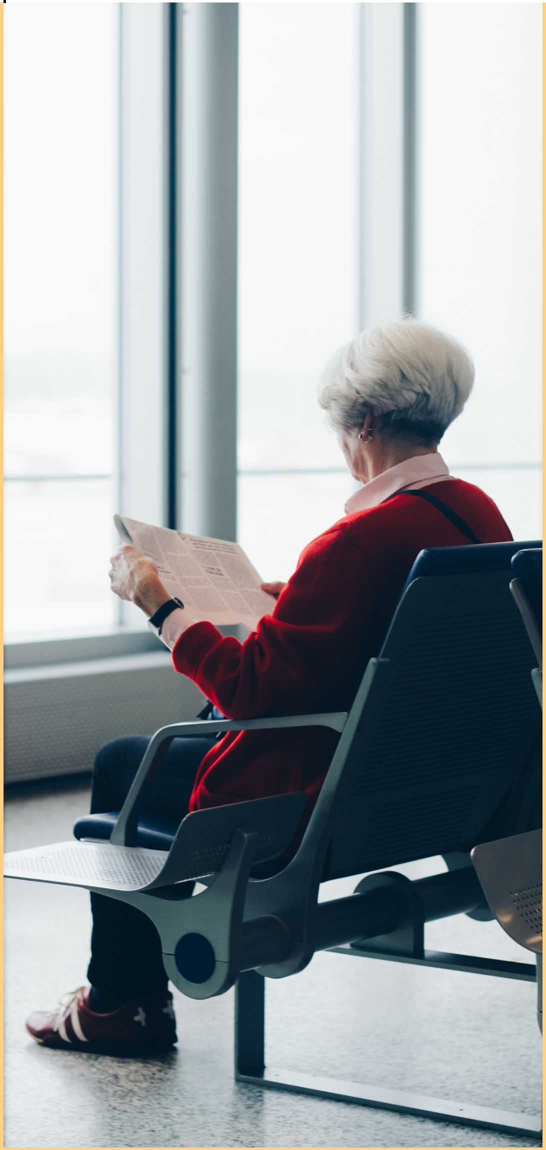

Think about your space

The environment can affect our behaviour. Think about:

- Where you are likely to sit for long periods of time?
- How could you change your surroundings to encourage you to sit less?

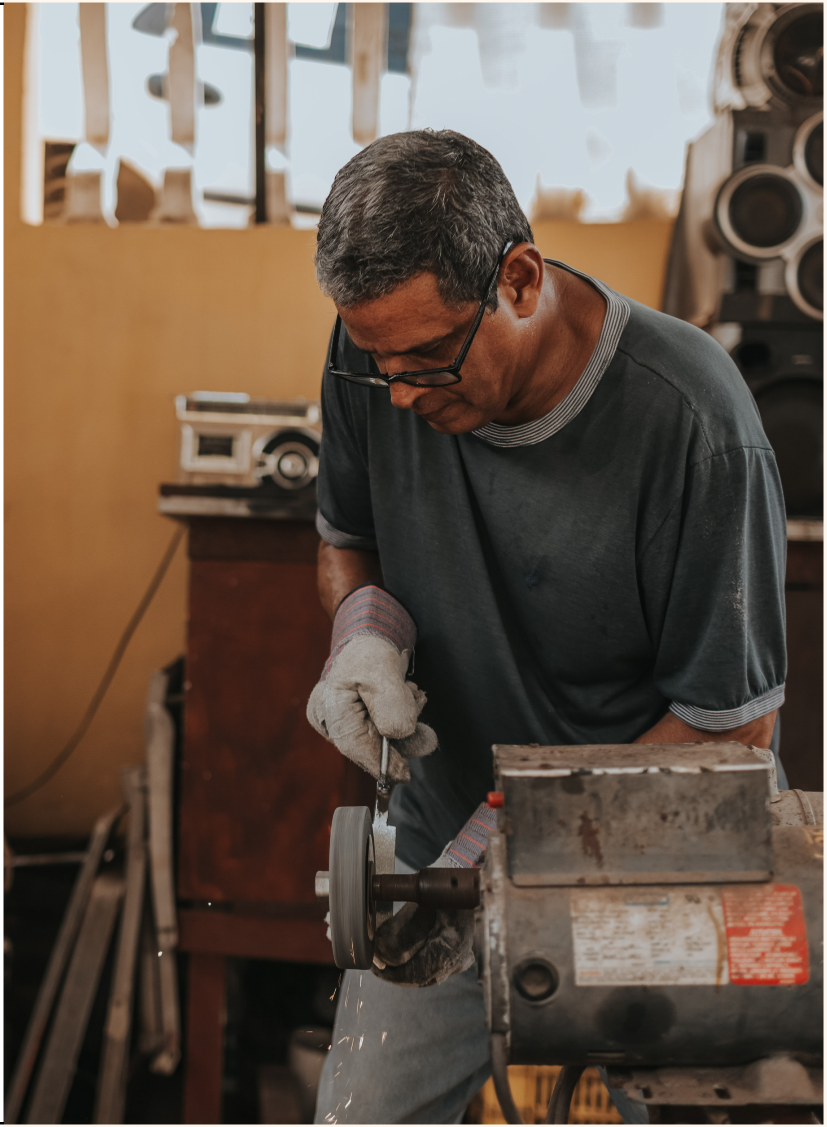

“I'm doing it to maintain my independence”

BUMPS IN THE ROAD

Sometimes changing our behaviour can be difficult. This task will help you plan how to avoid or cope with 'bumps in the road' and increase your chance of success. Think of difficult situations and how you might avoid/cope with them.

**IF ...**	**THEN...**
•	•
•	•
•	•

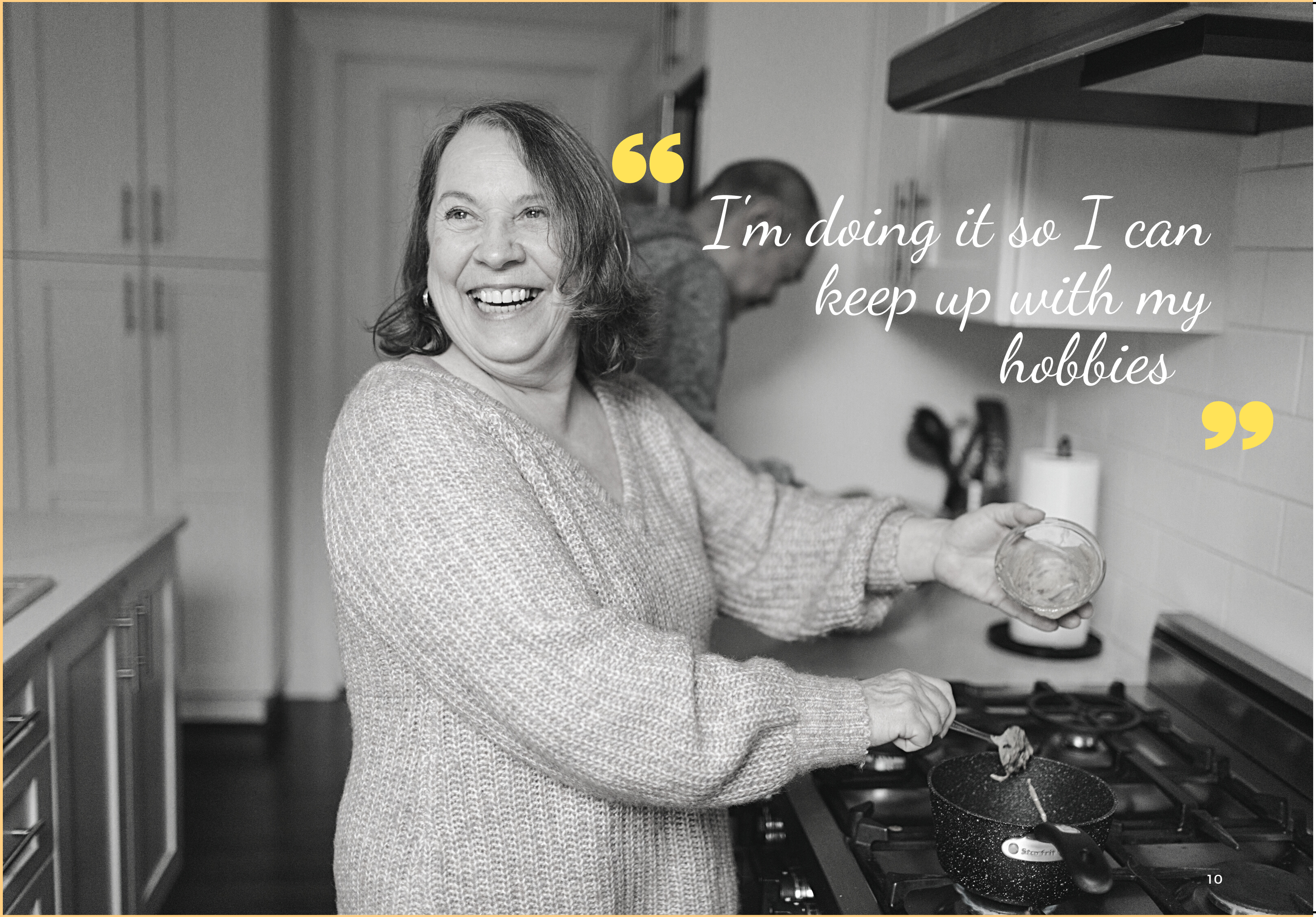

> “I'm doing it so I can keep up with my hobbies”

THIS IS WHERE YOUR PATH BEGINS

01

FEEDBACK & PERSONAL PLAN

GET FEEDBACK ON YOUR SITTING TIME

WORK THROUGH THIS BOOKLET TO SET GOALS AND MAKE ACTION PLANS

ASK QUESTIONS AND GET MOTIVATED

02

24-WEEK PROGRAMME

THE AIM OF THE PROGRAMME IS TO HELP YOU REDUCE YOUR SITTING TIME THROUGHOUT THE DAY

CREATE ACTION PLANS

SET THOSE PLANS IN MOTION

MONITOR YOUR PROGRESS WITH A WEARABLE DEVICE

BE SUPPORTED BY A HEALTH COACH TO MEET YOUR GOALS

03

LET'S GET STARTED!

BY COMPLETING THIS BOOKLET YOU'RE ALREADY ON THE PATH TO SUCCESS

TALK ABOUT YOUR WORKBOOK ANSWERS WITH YOUR HEALTH COACH

NOW GO ONE STEP FURTHER, PRACTICE STANDING AND MOVING FOR 2-3 MINUTES

Frail-LESS

Sit less.
Move more.
Live better.

QUESTIONS?

Email the research team:
Jamie.Harper@brunel.ac.uk
or
Daniel.Bailey@brunel.ac.uk

Or reach us by telephone:
01895 265035

Brunel University London

University of Bedfordshire

This consultation booklet is adapted from the Active Herts programme (www.activeherts.org.uk)

Active Herts
Your first step to a more active lifestyle
