# Supplementary material for: The frail-LESS (LEss sitting and sarcopenia in frail older adults) remote intervention to improve sarcopenia and maintain independent living via reductions in sedentary behaviour: findings from a randomised controlled feasibility trial
Source: BMC Geriatr. 2024 Sep 9;24:747. doi: 10.1186/s12877-024-05310-9 (PMC11382500; doi:10.1186/s12877-024-05310-9)
Supplement: Supplementary file 2 — Additional file 2 [file 12877_2024_5310_MOESM2_ESM.docx]

**Frail-LESS programme – Your baseline sitting time feedback**

Well done! You have already completed an important stage in your Frail-LESS programme, which aims to support you in breaking up and reducing how much time you spend sitting. You recently wore an activity monitor so we could measure your sitting time, and the purpose of this sheet is to give you feedback on your sitting and physical activity.

Here is a breakdown of how much of your day you spent sitting, standing and stepping:

On average you spent 11.2 hours sitting and did 4693 steps each day.

A break in sitting is when you get up to stand or move. On average, you took 47 breaks from sitting each day.

You can use this feedback to set goals for yourself around reducing and breaking up your sitting when going through your Frail-LESS workbook. You can also discuss this feedback with your health coach who will support you with sitting less. You will receive feedback again 3 and 6 months into the programme.
