# Supplementary material for: The frail-LESS (LEss sitting and sarcopenia in frail older adults) remote intervention to improve sarcopenia and maintain independent living via reductions in sedentary behaviour: findings from a randomised controlled feasibility trial
Source: BMC Geriatr. 2024 Sep 9;24:747. doi: 10.1186/s12877-024-05310-9 (PMC11382500; doi:10.1186/s12877-024-05310-9)
Supplement: Supplementary file 3 — Additional file 3 [file 12877_2024_5310_MOESM3_ESM.docx]

**Additional File 3**

Recruitment and eligibility information

| **Recruitment and eligibility details** | **n** | **%** |
| --- | --- | --- |
| GP recruitment |  |  |
| GP practices approached | 92 | - |
| GP practices participated | 52 | 57% |
| Patients identified | 13,755 | - |
| Patients contacted by text message | 11,910 | 87% |
| Expressions of interest | 239 | 2% |
| Other sources of recruitment |  |  |
| Lindus Health | 66 | - |
| Brunel Older People's Reference Group | 3 | - |
| Word of mouth | 3 | - |
| Total expressions of interest | 320 | - |
| Assessed for eligibility | 198 | 62% |
| Eligibility rate | 83 | 42% |
| Recruitment rate | 60 | 72% |

Retention rates at follow-up time points (n=60 participants recruited into the study).

|  |  |  |  |  |  |  |
| --- | --- | --- | --- | --- | --- | --- |
|  |  | **3 months** | |  | **6 months** | |
|  |  | **n** | **%** |  | **n** | **%** |
| All |  | 52 | 87 |  | 50 | 83 |
| Control |  | 27 | 90 |  | 26 | 87 |
| Intervention |  | 25 | 83 |  | 24 | 80 |

Descriptive statistics for falls and unplanned hospital and GP visits. Data presented as mean and SD.

|  | **Baseline** | | **3 Months** | | **6 Months** | | **Change (baseline to 3 months)** | | **Change (baseline to 6 months)** | |
| --- | --- | --- | --- | --- | --- | --- | --- | --- | --- | --- |
|  | **Control** | **Intervention** | **Control** | **Intervention** | **Control** | **Intervention** | **Control** | **Intervention** | **Control** | **Intervention** |
|  | (n = 30) | (n = 29) | (n = 26) | (n = 24) | (n = 26) | (n = 23) | (n = 26) | (n = 24) | (n = 26) | (n = 23) |
| Unplanned Hospital Visits in past 3 months^a^ | - | - | 0.12 | 0.04 | 0.04 | 0.22 | - | - | - | - |
|  |  |  | 0.43 | 0.20 | 0.20 | 0.42 | - | - | - | - |
| Unplanned GP Visits in past 3 months^a^ | - | - | 0.50 | 0.67 | 0.54 | 0.52 | - | - | - | - |
|  |  |  | 1.10 | 1.17 | 0.90 | 0.99 | - | - | - | - |
| Falls in past 3 months | 0.50 | 0.52 | 0.31 | 0.42 | 0.69 | 0.39 | -0.23 | 0.04 | 0.15 | 0.09 |
|  | 0.78 | 1.21 | 0.62 | 0.88 | 1.85 | 0.58 | 0.71 | 0.86 | 1.99 | 1.00 |

Change statistics were calculated only for participants who provided data at every time point.

**^a^**not assessed at baseline.

Descriptive statistics for SarQol. Data presented as mean and SD.

|  | **Baseline** | | **3 Months** | | **6 Months** | | **Change (baseline to 3 months)** | | **Change (baseline to 6 months)** | |
| --- | --- | --- | --- | --- | --- | --- | --- | --- | --- | --- |
|  | **Control** | **Intervention** | **Control** | **Intervention** | **Control** | **Intervention** | **Control** | **Intervention** | **Control** | **Intervention** |
|  | (n = 30) | (n = 29) | (n = 26) | (n = 24) | (n = 26) | (n = 23) | (n = 26) | (n = 24) | (n = 26) | (n = 23) |
| Overall SarQol | 57.6 | 59.3 | 63.5 | 63.4 | 63.9 | 65.3 | 6.2 | 4.8 | 6.0 | 6.5 |
|  | 13.2 | 11.2 | 14.5 | 11.3 | 14.3 | 14.8 | 8.5 | 7.0 | 8.2 | 13.1 |
| D1 Physical and Mental Health | 57.8 | 62.9 | 68.1 | 69.7 | 69.3 | 69.7 | 11.7 | 8.1 | 12.0 | 6.7 |
|  | 15.2 | 14.0 | 16.6 | 14.1 | 16.2 | 15.6 | 13.2 | 16.1 | 15.0 | 18.5 |
| D2 Locomotion | 53.9 | 49.4 | 57.3 | 52.0 | 59.8 | 57.4 | 3.2 | 3.1 | 6.0 | 8.1 |
|  | 15.7 | 14.1 | 20.0 | 15.5 | 17.1 | 16.9 | 15.7 | 11.7 | 8.2 | 17.7 |
| D3 Body Composition | 56.1 | 60.6 | 66.6 | 61.6 | 65.1 | 65.8 | 11.5 | 3.4 | 10.3 | 5.8 |
|  | 13.7 | 16.6 | 16.1 | 11.2 | 16.8 | 15.2 | 16.1 | 14.1 | 16.6 | 17.5 |
| D4 Functionality | 63.0 | 65.1 | 68.0 | 69.9 | 68.7 | 71.8 | 5.1 | 4.6 | 4.8 | 6.2 |
|  | 15.1 | 12.3 | 13.9 | 15.6 | 15.8 | 14.9 | 10.2 | 8.0 | 10.4 | 12.4 |
| D5 Activities of daily living | 52.7 | 57.7 | 58.4 | 60.6 | 57.0 | 60.7 | 6.0 | 4.0 | 3.5 | 5.1 |
|  | 17.9 | 15.0 | 18.7 | 15.1 | 16.7 | 18.5 | 10.2 | 11.9 | 9.6 | 15.2 |
| D6 Leisure activities | 46.0 | 37.8 | 51.8 | 47.8 | 54.4 | 54.2 | 4.5 | 9.0 | 7.0 | 15.2 |
|  | 22.1 | 14.0 | 27.6 | 19.8 | 27.2 | 26.6 | 29.9 | 18.3 | 31.3 | 24.5 |
| D7 Fears | 85.0 | 82.8 | 87.5 | 85.9 | 87.5 | 87.0 | 1.9 | 3.6 | 2.9 | 3.3 |
|  | 12.0 | 13.5 | 10.6 | 12.9 | 13.2 | 14.8 | 13.1 | 13.5 | 12.4 | 13.7 |

Change statistics were calculated only for participants who provided data at every time point.

SarQol items are rated on a 4-point Likert scale. Each item is scored between 0 and 100. The scoring algorithm is available from the questionnaire authors <https://www.sarqol.org/en>. Higher scores reflect higher quality of life.

Descriptive statistics for self-reported physical function, mood and wellbeing. Data presented as mean and SD.

|  | **Baseline** | | **3 Months** | | **6 Months** | | **Change (baseline to 3 months)** | | **Change (baseline to 6 months)** | |
| --- | --- | --- | --- | --- | --- | --- | --- | --- | --- | --- |
|  | **Control** | **Intervention** | **Control** | **Intervention** | **Control** | **Intervention** | **Control** | **Intervention** | **Control** | **Intervention** |
|  | (n = 30) | (n = 29) | (n = 26) | (n = 24) | (n = 26) | (n = 23) | (n = 26) | (n = 24) | (n = 26) | (n = 23) |
| SARC-F | 2.5 | 2.6 | 2.2 | 2.3 | 2.2 | 2.1 | -0.3 | -0.2 | -0.2 | -0.4 |
|  | 1.7 | 1.9 | 1.7 | 1.6 | 2.0 | 1.8 | 1.4 | 1.0 | 1.3 | 1.4 |
| GARS | 25.5 | 24.2 | 24.8 | 23.3 | 23.3 | 22.5 | -0.2 | -1.2 | -1.4 | -2.1 |
|  | 8.3 | 6.5 | 9.2 | 6.6 | 5.9 | 5.8 | 3.3 | 2.8 | 4.2 | 3.2 |
| Positive Affect | 26.7 | 31.3 | 26.5 | 32.8 | 28.0 | 32.3 | 0.0 | 2.5 | 0.7 | 2.8 |
|  | 9.5 | 6.8 | 8.1 | 8.3 | 10.3 | 9.2 | 7.2 | 5.3 | 8.0 | 6.5 |
| Negative Affect | 16.3 | 16.8 | 14.8 | 14.8 | 15.3 | 15.8 | -1.3 | -1.1 | -1.0 | -0.7 |
|  | 6.5 | 6.3 | 4.9 | 4.6 | 5.8 | 5.7 | 6.5 | 3.4 | 4.9 | 3.2 |
| ONS Life satisfaction | 6.8 | 6.4 | 6.1 | 7.1 | 6.2 | 7.1 | -0.7 | 0.6 | -0.8 | 0.8 |
|  | 2.3 | 2.0 | 2.6 | 1.7 | 2.7 | 2.1 | 2.1 | 1.1 | 2.1 | 1.3 |
| ONS Worthwhile | 6.8 | 7.4 | 6.7 | 7.4 | 7.1 | 7.7 | -0.1 | 0.0 | 0.2 | 0.4 |
|  | 2.3 | 1.9 | 2.6 | 1.6 | 2.4 | 2.2 | 2.2 | 1.2 | 2.1 | 1.3 |
| ONS Happiness | 6.7 | 6.8 | 6.7 | 7.5 | 6.8 | 7.3 | -0.1 | 0.5 | -0.2 | 0.5 |
|  | 2.3 | 2.2 | 2.6 | 2.1 | 2.5 | 2.2 | 2.2 | 2.0 | 2.4 | 1.9 |
| ONS Anxiety | 2.5 | 3.5 | 2.5 | 2.6 | 3.6 | 2.2 | 0.0 | -1.0 | 1.2 | -1.5 |
|  | 2.4 | 2.4 | 2.7 | 2.7 | 3.3 | 2.5 | 3.4 | 2.0 | 4.2 | 3.1 |
| Pain | 33.5 | 24.2 | 39.7 | 27.9 | 34.6 | 21.1 | 5.2 | 2.9 | 1.8 | -5.4 |
|  | 26.8 | 24.3 | 29.5 | 28.2 | 27.8 | 26.2 | 22.7 | 31.1 | 25.0 | 29.3 |
| Fatigue Severity Scale | 4.2 | 4.2 | 4.0 | 3.5 | 4.0 | 3.6 | -0.3 | -0.5 | -0.2 | -0.5 |
|  | 1.5 | 1.3 | 1.6 | 1.3 | 1.8 | 1.4 | 1.4 | 1.4 | 1.2 | 1.5 |

Change statistics were calculated only for participants who provided data at every time point for that outcome.

SARC-F, Strength, assistance with walking, rising from a chair, climbing stairs, and falls; GARS, Groningen Activity Restriction Scale; ONS, Office for National Statistics.

SARC-F – five items rated on a scale from 0 to 2 with scores summed. Higher scores reflect increased sarcopenia (SARC-F ≥ 4 is defined as sarcopenia).

GARS – 18 items rated on a scale of 1 (fully independent) to 4 (cannot do it fully independently) with scores summed. Higher scores reflect increased difficulty with activities of daily living.

Positive and Negative Affect – 10 items for positive and 10 items for negative affect each rated on a 5-point Likert Scale from “Very slightly or not at all” to “Extremely”. Scores are summed separately for each positive and negative affect with higher scores reflecting higher affect.

ONS – four items rated separately on a 10-point scale. Higher scores for Life satisfaction, Worthwhile and Happiness reflect higher wellbeing. Higher scores for Anxiety reflect lower wellbeing.

Pain – scored on a 100 mm visual analogue scale from “No pain” to “Worst pain imaginable”.

Fatigue Severity Scale – nine items scored on a 7-point Likert scale from “Strongly Disagree” to “Strongly Agree”. Arithmetic mean of all items is calculated with higher scroes reflecting higher fatigue (≥4 indicates severe fatigue).
