# Supplementary material for: The frail-LESS (LEss sitting and sarcopenia in frail older adults) remote intervention to improve sarcopenia and maintain independent living via reductions in sedentary behaviour: findings from a randomised controlled feasibility trial
Source: BMC Geriatr. 2024 Sep 9;24:747. doi: 10.1186/s12877-024-05310-9 (PMC11382500; doi:10.1186/s12877-024-05310-9)
Supplement: Supplementary file 4 — Additional file 4 [file 12877_2024_5310_MOESM4_ESM.docx]

**Additional file 4.** Effects of the study measurements on behaviour in the control and intervention groups.

|  |  | **3 Months** | | | | **6 Months** | | | |
| --- | --- | --- | --- | --- | --- | --- | --- | --- | --- |
|  |  | **Control (n = 26)** | | **Intervention (n = 24)** | | **Control (n = 26)** | | **Intervention (n = 23)** | |
| In the past 3 months has anything in your life changed that has had an impact on your health-related behaviours? | *Yes* | 11 | 42% | 14 | 58% | 10 | 38% | 15 | 65% |
|  | *No* | 15 | 58% | 10 | 42% | 16 | 62% | 8 | 35% |
| Do you feel being a part of the Frail-LESS study, despite being in the control group has changed your sitting behaviour over the past 3 months? | *Yes* | 9 | 35% | - | - | 12 | 46% | - | - |
|  | *No* | 17 | 65% | - | - | 14 | 54% | - | - |
| The offer of the testing session measurements encouraged me to participate in the study | *Strongly agree* | 5 | 19% | 13 | 54% | 5 | 19% | 7 | 30% |
|  | *Agree* | 11 | 42% | 7 | 29% | 13 | 50% | 10 | 43% |
|  | *Neither agree nor disagree* | 7 | 27% | 4 | 17% | 6 | 23% | 5 | 22% |
|  | *Disagree* | 0 | 0% | 0 | 0% | 1 | 4% | 0 | 0% |
|  | *Strongly disagree* | 3 | 12% | 0 | 0% | 1 | 4% | 1 | 4% |
| The testing sessions motivated me to want to change how much time I spent sitting | *Strongly agree* | 7 | 27% | 10 | 42% | 6 | 23% | 8 | 35% |
|  | *Agree* | 7 | 27% | 10 | 42% | 9 | 35% | 11 | 48% |
|  | *Neither agree nor disagree* | 8 | 31% | 4 | 17% | 7 | 27% | 3 | 13% |
|  | *Disagree* | 2 | 8% | 0 | 0% | 2 | 8% | 0 | 0% |
|  | *Strongly disagree* | 2 | 8% | 0 | 0% | 2 | 8% | 1 | 4% |
| Knowing that I would receive a follow up testing session motivated me to want to change aspects of my lifestyle behaviours | *Strongly agree* | 5 | 19% | 10 | 42% | 6 | 23% | 7 | 30% |
|  | *Agree* | 9 | 35% | 8 | 33% | 6 | 23% | 12 | 52% |
|  | *Neither agree nor disagree* | 8 | 31% | 5 | 21% | 10 | 38% | 3 | 13% |
|  | *Disagree* | 2 | 8% | 1 | 4% | 2 | 8% | 0 | 0% |
|  | *Strongly disagree* | 2 | 8% | 0 | 0% | 2 | 8% | 1 | 4% |
| Knowing that I would receive a follow up testing session motivated me to want to change how much time I spent sitting | *Strongly agree* | 9 | 35% | 11 | 46% | 5 | 19% | 10 | 43% |
|  | *Agree* | 3 | 12% | 8 | 33% | 10 | 38% | 9 | 39% |
|  | *Neither agree nor disagree* | 10 | 38% | 4 | 17% | 8 | 31% | 3 | 13% |
|  | *Disagree* | 2 | 8% | 1 | 4% | 1 | 4% | 0 | 0% |
|  | *Strongly disagree* | 2 | 8% | 0 | 0% | 2 | 8% | 1 | 4% |

% calculated as number of responses / number of participants that fully completed this set of questionnaire items at each timepoint x 100.
